# Supplementary material for: Homogeneous Zero-Index Thermal Metadevice for Thermal Camouflaging and Super-Expanding
Source: Materials (Basel). 2023 May 11;16(10):3657. doi: 10.3390/ma16103657 (PMC10221638; doi:10.3390/ma16103657)
Supplement: Supplementary file 1 [file materials-16-03657-s001.zip › materials-2260178-supplementary.pdf]

materials

Supporting Information

Homogeneous Zero-index Thermal Metadevice for Thermal Camouflaging and Super-expanding

Huagen Li [1,†], Kaipeng Liu [1,†], Tianfeng Liu [2,†] and Run Hu *

[1] Department of Electrical and Computer Engineering, National University of Singapore, Singapore 117583, Singapore; e0383074@u.nus.edu (H.L.); kaipengliu@yeah.net (K.L.)
[2] State Key Laboratory of Coal Combustion, School of Energy and Power Engineering, Huazhong University of Science and Technology, Wuhan 430074, China; m202271354@hust.edu.cn
* Correspondence: hurun@hust.edu.cn
† These authors contributed equally to this work.

Supplementary Note S1: The relation of effective thermal conductivity and the central ultra-thin wall with hollow structure

Firstly, as shown in Figure S1a and S1b, the actual thermal conductivities of the ultra-thin hollow wall and background are κ_u and κ_b, respectively. The thickness of the background and the ultra-thin hollow wall are correspondingly denoted by d and σ. Given that the thickness σ is quite small, we can consider there is an effective heat source along the bottom boundary of this ultra-thin hollow wall and the average effective heat source in the thickness d of the background is denoted by Q_v^{eff} [1]. Thus, $Q_v^{eff} = q_u/d$ and q_u should possess the terms of $\cos\theta$. We can assume $q_u/d = U\cos\theta$ with an undetermined U. Then, the general solutions of temperature for this structure based on the steady-state heat conduction equation are expressed as follows [1]:

$$T_1 = \left(-\frac{U}{3\kappa_u}r^2 + A_1 r + B_1 r^{-1}\right)\cos\theta \quad \left(r_u - \sigma < r \le r_u\right) \tag{S1}$$

$$T_2 = \left(A_2 r + B_2 r^{-1}\right)\cos\theta\left(r > r_u\right) \tag{S2}$$

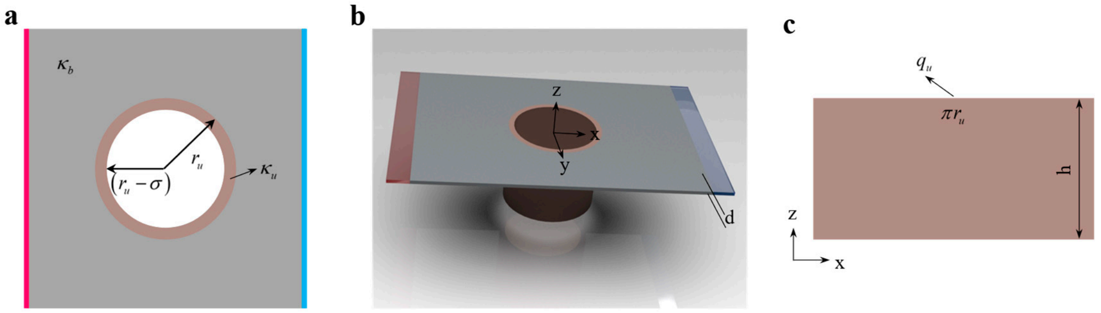

Figure S1. 2D (a) and 3D (b) Schematic of the central ultra-thin hollow wall structure embedded in the background. (c) Unfolded ultra-thin hollow wall.

The boundary conditions can be written as:

$$\begin{cases} -\dfrac{U}{3\kappa_b}r_u^2 + A_1 r_u + B_1 r_u^{-1} = A_2 R_u + B_2 r_u^{-1} \\ \kappa_u\left(-\dfrac{2U}{3\kappa_u}r_u + A_1 - B_1 r_u^{-2}\right) = \kappa_b\left(A_2 - B_2 r_u^{-2}\right) \end{cases} \left(r = r_u\right) \tag{S3}$$

where A and B in Equation (S2) and (S3) are undetermined parameters of the solutions, which can be derived based on the continuity of the boundary conditions at each interface.

If we consider an effective 2D model is equivalent to the current 3D model with an ultra-thin hollow wall, the corresponding effective region of the ultra-thin hollow wall possesses a thermal conductivity κ_u^{eff}. Then, the general solutions for the temperature of each effective region can be shown as follows:

$$T_1^{eff} = \left(C_1 r + D_1 r^{-1}\right)\cos\theta \quad \left(r_u - \sigma < r \le r_u\right) \tag{S4}$$

$$T_2^{eff} = \left(C_2 r + D_2 r^{-1}\right)\cos\theta\left(r > r_2\right) \tag{S5}$$

The above C and D are determined by the boundary conditions as follows:

$$\begin{cases} C_1 r_u + D_1 r_u^{-1} = C_2 R_2 + D_2 r_u^{-1} \\ \kappa_u^{\mathrm{eff}}\left(C_1 - D_1 r_u^{-2}\right) = \kappa_b\left(C_2 - D_2 r_u^{-2}\right) \end{cases} \left(r = r_u\right) \tag{S6}$$

Since the background in the actual model and effective model should be equivalent, the B_2 should be equal to D_2. Further, the ultra-thin hollow wall is unfolded to a 2D plane (Figure S1c). The temperature distributions at z = 0 should use the temperature distributions at the outer boundary $\left(r = r_u\right)$ of the ultra-thin hollow wall, written as follows:

$$T_u^{outer}\Big|_{z=0} = \left(A_2 r_u + B_2 r_u^{-1}\right)\cos\frac{x}{r_u} \tag{S7}$$

Then, the inner temperature distributions of the ultra-thin hollow wall can be expressed as:

$$T_u^{inner}\Big|_{z=0} = V\left(r_u - \sigma\right)\cos\frac{x}{r_u - \sigma} \tag{S8}$$

Since other boundaries of the opened ultra-thin hollow wall are thermal insulations, the general solution of such conditions can be described as [1]:

$$T_u^{inner} = \sum_{n=1}^{\infty} W_n \cos\left(\frac{n}{r_u - \sigma}x\right)\cosh\left(\frac{n}{r_u - \sigma}\left(h - z\right)\right) \tag{S9}$$

From Equation (S8) and (S9), we can obtain the equal relation of T_u^{inner} at z = 0 as follows:

$$V\left(r_u - \sigma\right)\cos\frac{x}{r_u - \sigma} = \sum_{n=1}^{\infty} W_n \cos\left(\frac{n}{r_u - \sigma}x\right)\cosh\left(\frac{n}{r_u - \sigma}h\right) \tag{S10}$$

Then, the general expression of W_n can be derived as follows:

$$W_n = \frac{V\left(r_u - \sigma\right)\int_0^{\pi\left(r_u - \sigma\right)} \cos\frac{x}{r_u - \sigma}\cos\left(\frac{n}{r_u - \sigma}x\right)dx}{\cosh\left(\frac{n}{r_u - \sigma}h\right)\int_0^{\pi\left(r_u - \sigma\right)} \cos^2\left(\frac{n}{r_u - \sigma}x\right)dx} \tag{S11}$$

According to the temperature distributions at z = 0, the explicit form of W_n can be acquired as follows:

$$\begin{cases} W_1 = \dfrac{V\left(r_u - \sigma\right)}{\cosh\left(\dfrac{h}{r_u - \sigma}\right)} \\ W_n = 0, n \ne 1 \end{cases} \tag{S12}$$

Thus, the temperature expression of the T_u^{inner} is:

$$T_u^{inner} = \frac{V\left(r_u - \sigma\right)}{\cosh\left(\frac{h}{r_u - \sigma}\right)} \cos\left(\frac{x}{r_u - \sigma}\right) \cosh\left(\frac{h - z}{r_u - \sigma}\right) \tag{S13}$$

According to the ultra-thin structure of the hollow wall, the heat flux at the inner boundary is determined by

$$q_u = -\kappa_u \left.\frac{\partial T_u^{inner}}{\partial z}\right|_{z=0} = -\kappa_u V \cos\left(\frac{x}{r_u - \sigma}\right) \tanh\left(\frac{h}{r_u - \sigma}\right) \tag{S14}$$

Since $q_u / d = U \cos\theta$, we can calculate constant U as:

$$U = -\frac{V\kappa_u}{d} \tanh\left(\frac{h}{r_u - \sigma}\right) \tag{S15}$$

Here, combining equations (S3), (S6) and (S16), we can derive the expression of κ_u^{eff} as follows:

$$k_u^{eff} = \frac{-k_u m_2 \tanh\left[h/\left(r_u - \sigma\right)\right] + 3k_b m_1 \left(r_u - \sigma\right)}{3m_1 d\left(r_u - \sigma\right)} \tag{S16}$$

The m_1 and m_2 are related with r_u and σ, as

$$m_1 = r_u^2 - \left(r_u - \sigma\right)^2 \tag{S17}$$

$$m_2 = r_u^4 + 3\left(r_u - \sigma\right)^2 r_u^2 - 4\left(r_u - \sigma\right)^3 r_u \tag{S18}$$

where κ_u and κ_b are the corresponding thermal conductivities of the ultra-thin elliptical hollow wall and background. Then, r_u and h is the radius and height of the ultra-thin hollow wall. d and σ are the thickness of the ultra-thin hollow wall and background, respectively. Herein, it is easy to see that the background temperature fields vary from convergence to divergence with the increase of heights of the ultra-thin hollow wall as shown in Figure S2.

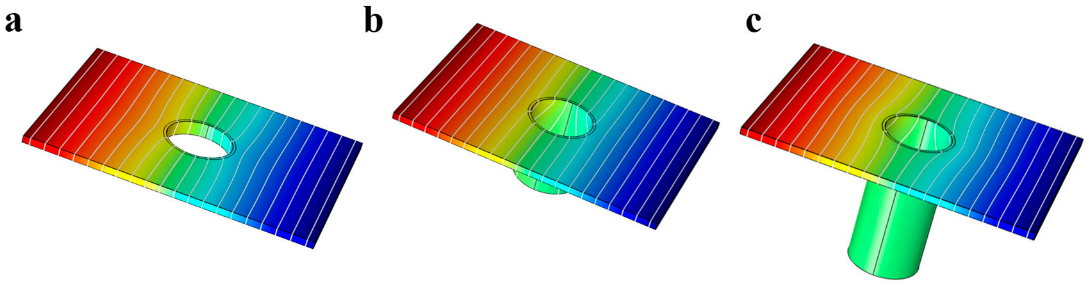

Figure S2. The temperature fields of the whole structure when the heights (*h*) of this ultra-thin hollow wall are (**a**) 2 mm, (**b**) 6 mm and (**c**) 20 mm, respectively.

Supplementary Note S2: The relation about the original temperature and on-demand new temperature value of the thermostatic boundary due to a shift of the ultra-thin hollow wall from the background center

If we introduce the temperature modulation as another strategy to acquire the homogeneous zero-index scheme, as shown in Figure S3a, we can obtain the constant temperature for this ultra-thin hollow wall based on the steady-state heat conduction equation. Now let's assume this constant temperature to T_u^{ori}. Thus, when this ultra-thin hollow

wall moves left or right with a value Δx (assume the left shift is a negative value), the constant temperature should need a corresponding change α to realize an IETC for this ultra-thin hollow wall.

$$T_u^{new} = T_u^{ori} + \alpha \tag{S19}$$

$$\alpha = \frac{\Delta T}{L}\left(\varepsilon' + \Delta x\right)\left(\left|\Delta x\right| \le 0.5L\right) \tag{S20}$$

As shown in Figure S3b and 3c, no matter how we change the value of Δx in the background with a length *L*, we find out the temperature curves of the whole structure at the line (y = 0 mm) always passes one point which also deviates a value ε' in contrast to the center of ultra-thin hollow wall. Thus, there is a size shift effect in this structure and the relation is expressed as follows:

$$\frac{r_u - \varepsilon'}{0.5L - \Delta x} = \frac{r_u}{0.5L} \tag{S21}$$

$$\varepsilon' = \frac{r_u}{0.5L}\Delta x \tag{S22}$$

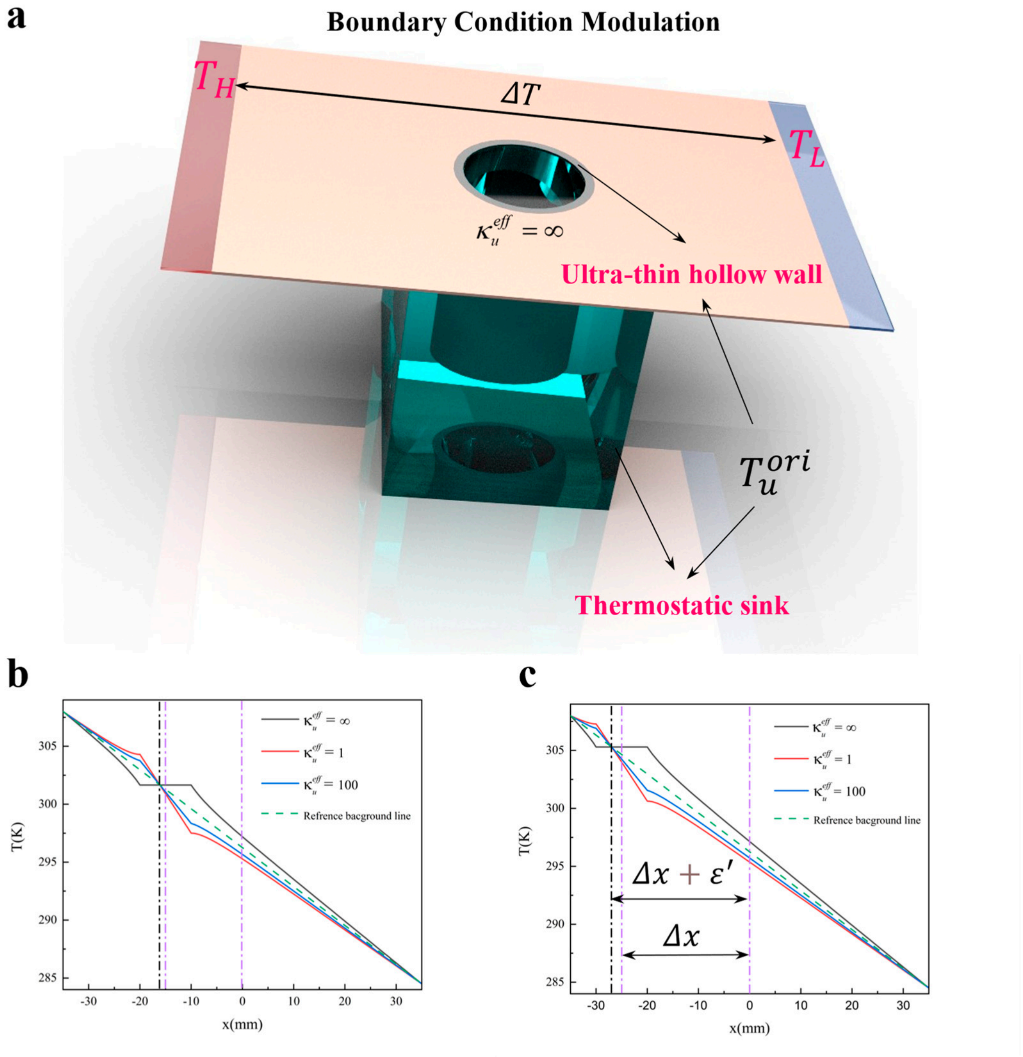

Figure S3. (a) 3D Schematic of the ultra-thin hollow wall structure embedded in the background. (b) and (c) the temperature curves of the whole structure at the line (y = 0 mm) when Δx takes two different values.

Thus, combining equations (S19), (S20) and (S22), we can derive the temperature on-demand new temperature value T_u^{ori} as follows:

$$T_u^{new} = T_u^{ori} - \frac{L+2r_u}{L^2}\Delta T \Delta x \left(|\Delta x| \le 0.5L\right) \tag{S23}$$

Supplementary Note S3: The comparison between our homogeneous zero-index scheme and the rotating near-zero-index scheme

1) The rotating scheme [2, 3] has been proposed to achieve the near infinite effective thermal conductivity (IETC) and adjustable ETC by rotating fluids. Such a rotating scheme is quite interesting and can be used to achieve large ETC beyond naturally occurring materials. But the rotating scheme cannot exactly obtain the infinite effective thermal conductivity in practice. Then, based on the above discussions, we propose and design our homogeneous zero-index scheme beyond rotating to realize the IETC and its effective thermal conductivity can be highly tuned by changing the height of the ultra-thin wall with a new theoretical framework.

2) Then, the rotating fluid scheme needs a highly spinning motor, which consumes massive energy input, while our current scheme needs low energy input.

3) Due to rotating with the same angular velocity, the linear velocity of the metadevice is high, resulting in the unavoidable phenomena that the ETC along the tangential direction is very large while the ETC along the radial direction is small. As a result, it is hard to achieve thermal camouflage and thermal expander functionalities by this rotating scheme. However, our scheme can flexibly and easily realize the zero-index thermal camouflage device and zero-index super thermal expander, as demonstrated in our paper theoretically and experimentally.

In all, our homogeneous zero-index scheme is quite robust and feasible to fabricate, and it will further provide more robust and flexible zero-index thermal metadevices. The advantage comparison of our homogeneous zero-index scheme is shown in Figure S4.

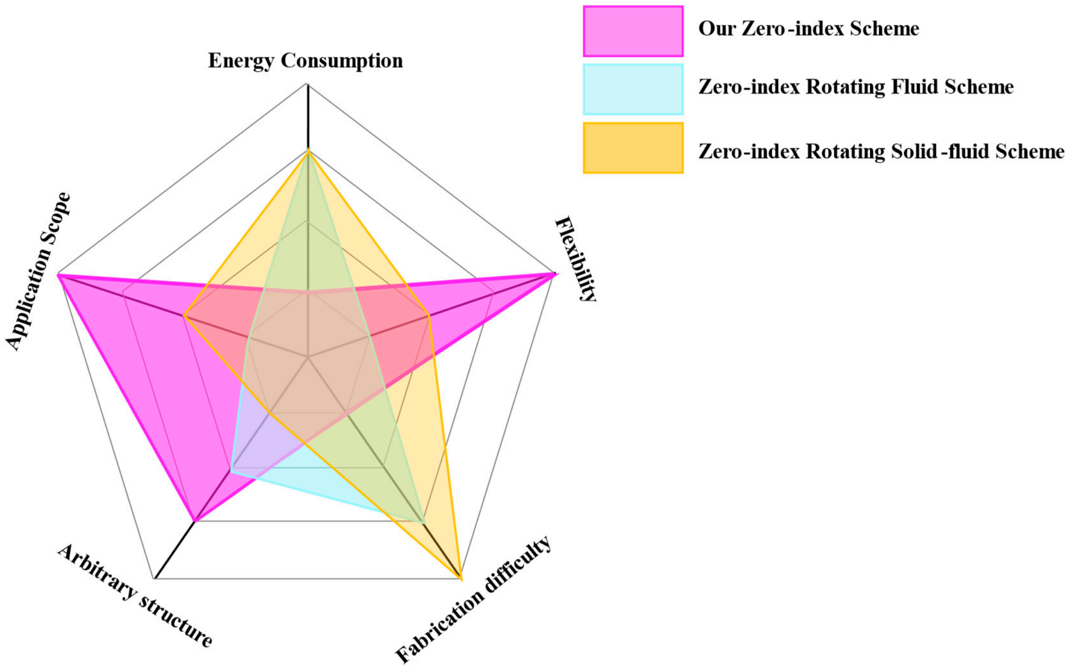

Figure S4. Comparison of these schemes for controlling heat flow in terms of energy consumption, application scope, arbitrary structure, fabrication difficulty, and flexibility.

Supplementary References

1. J. Guo, G. Xu, D. Tian, Z. Qu, C.-W. Qiu, Passive Ultra-Conductive Thermal Metamaterials, Adv. Mater. 34 (2022) 2200329. https://doi.org/10.1002/adma.202200329.
2. Y. Li, K.-J. Zhu, Y.-G. Peng, W. Li, T. Yang, H.-X. Xu, H. Chen, X.-F. Zhu, S. Fan, C.-W. Qiu, Thermal meta-device in analogue of zero-index photonics, Nat. Mater. 18 (2019) 48–54. https://doi.org/10.1038/s41563-018-0239-6.
3. G. Xu, K. Dong, Y. Li, H. Li, K. Liu, L. Li, J. Wu, C.-W. Qiu, Tunable analog thermal material, Nat. Commun. 11 (2020) 6028. https://doi.org/10.1038/s41467-020-19909-0.
